# Pulsating aerosols for the rhinosinusitis treatment – evaluation of an innovative method in a multicentric survey

**DOI:** 10.1186/2045-7022-3-S2-P38

**Published:** 2013-07-16

**Authors:** Heribet W Mentzel, Rosina Ledermueller, Kirsteen Watt

**Affiliations:** 1 PARI GmbH, Starnberg, Germany; 2 PARI GmbH, Medical Affairs, Starnberg, Germany; 3 PARI GmbH, International Marketing, Starnberg, Germany

## Background

Acute and Chronic Rhinosinusitis (ARS/CRS) have a strong health- economic impact. It can be assumed that 10-15% of the US and EU population suffers from CRS. The success of topical drug delivery to the sinuses - principally desirable - is limited because the paranasal cavities are virtually non-ventilated areas. An innovative inhalation system (PARI SINUS) which provides a pulsating aerosol has been shown to improve the particle deposition within the paranasal sinuses compared to an inhalation without vibration. Patients' acceptance of this device and the clinical effects of the therapy have been evaluated.

## Method

In this multicentric, non-interventional, retrospective survey, private ENT-specialists completed a 2-page-questionnaire with data from patients who received inhalation therapy with saline solution via the pulsating aerosol. Most of the parameters were assessed on a 7-point scale from -3 (very negative) to +3 (very positive).

## Results

The data from 33 ARS patients (17 female (f), 16 male (m) average age 39,3 **±**18 and 48 CRS patients (29 f, 22 m, average age 49,8 **±**12) who received the treatment twice a day (mean 2,05 **±**0,92) were evaluated. The acceptance of the therapy and the handling of the inhalation technique were rated as +2,46 **±**0,95 and +2,27 **±**0,98 respectively. The general effect on symptoms was valued +2,27 **±**0,36 for ARS and +1,76 **±**0,43 for CRS patients. The impact on quality of life (QOL) was graded +2,39 **±**0,84 for ARS and +2,18 **±**0,76 for CRS. The course of the disease +2,48 **±**0,81 (ARS) and +2,05 **±**1,01 (CRS). For ARS patients the therapy led to a reduction in the use of nasal steroids (+1,42 **±**1,26) and oral antibiotics (+1,63 **±**1,31). CRS patients also needed fewer nasal steroids (+1,19 **±**1,19) and oral antibiotics (+1,49 **±**1,06). A decrease in sick leave days was reported for both patient groups (ARS +1,70 **±**1,07, CRS +1,26 **±**1,23).

## Conclusions

Inhalation therapy for the paranasal sinuses via pulsating aerosol is well accepted by ARS and CRS patients. Substantial symptom relief, improvement of QOL, lowering of the need for oral antibiotics and nasal steroids as well as the decline in sick leave days make this non-invasive and painless therapy an interesting treatment option. Further studies for ARS and CRS are desirable to investigate the full potential of this topical therapy option.

